# Prevalence of cognitive impairment (CI) in patients with multiple sclerosis (MS): A systematic review and meta-analysis 

**DOI:** 10.22088/cjim.15.3.392

**Published:** 2024-08-01

**Authors:** Mozhde Askari, Omid Mirmosayyeb, Fatemeh Fattahi, Hamed Ghoshouni, Elham Moases Ghaffary, Vahid Shaygannejad, Mahsa Ghajarzadeh

**Affiliations:** 1 Isfahan Neurosciences Research Center, Isfahan University of Medical Sciences, Isfahan, Iran; 2 Multiple Sclerosis Research Center, Neuroscience Institute, Tehran University of Medical Sciences, Tehran, Iran; 3 Universal council of epidemiology (UCE), Universal Scientific Education and Research Network (USERN), Tehran, Iran

**Keywords:** Multiple sclerosis, Cognitive impairment, Systematic review

## Abstract

**Background:**

One of the complications of multiple sclerosis (MS) is cognitive impairment (CI). The prevalence of CI is reported variously in previous studies. The goal of this systematic review and meta-analysis to estimate pooled prevalence of CI in patients with MS and also the prevalence of CI based on the type of applied test.

**Methods:**

Two independent researchers systematically searched PubMed, Scopus, EMBASE, Web of Science, and google scholar as well as gray literature (conference abstracts, references of the references) which were published before up January 2022.

**Results:**

We found 4089 articles by literature search, after deleting duplicates 3174 remained. Ninety articles remained for meta-analysis. The pooled prevalence of CI using all types of tests was 41% (95% CI: 38-44%) (I2=91.7%, p<0.001). The pooled prevalence of CI using BRB test was 39% (95%CI: 36-42%) (I2=89%, p<0.001). The pooled prevalence of CI using BICAMS was 44% (95%CI: 37-51%, I2=95.4%, p<0.001). The pooled prevalence of CI using MACFIMS was 44% (95% CI: 36-53%) (I2=89.3%, p<0.001).

**Conclusions:**

The pooled prevalence of cognitive impairment in patients with MS is estimated as 41%, so CI it should be considered by clinicians.

Multiple sclerosis (MS), an autoimmune disease of central nervous system (CNS) has lots of physical and psychological complications (1, 2). Cognitive impairment (CI) is one of the disabling complications of MS affecting between 40-65% of patients with MS (4). It has negative impacts on daily activities, social functioning, employment, education continuation, and finally the total quality of life (5). CI could be detected from earlier stages and progress during the time (6). CI is more prominent and more domains of cognition are affected in patients with progressive form of the disease (7). There is heterogeneity regarding degree and scope of CI in MS while the most common deficit is slowing of information processing speed and learning/memory inefficiency (8).

Different tests such as Paced Auditory Serial Addition Test (PASAT), Symbol Digit Modalities Test (SDMT), Brief Repeatable Battery (BRB), minimal assessment of cognitive function in multiple sclerosis (MACFIMS) or its brief form (BICAMS) are applied for cognitive assessment in patients with MS(9). Each test evaluates different aspects of cognition and has its own advantages and disadvantages.

Up to now, lots of studies reported prevalence of cognitive impairment using different tests, but the pooled prevalence of CI based on different available tests are not present. So, we designed this systematic review and meta-analysis to estimate pooled prevalence of CI in patients with MS and also the prevalence of CI based on the type of applied test.

## Methods

**Study design:** Systematic review, and meta-analysis. Two independent researchers systematically searched PubMed, Scopus, EMBASE, Web of Science, and Google scholar as well as gray literature (conference abstracts, references of the references) which were published before up January 2022. The search was done on January 1st 2022.

The search terns was: (“Multiple Sclerosis” OR “MS” OR “Relapsing-Remitting Multiple Sclerosis” OR “Chronic Progressive Multiple Sclerosis” OR “demyelinating diseases” OR “demyelinating disorders” OR “autoimmune demyelinating disease" AND “Cognitive Behavior Therapy” OR “Cognitive Therapy” OR “Cognitive Behavior Therapy” OR “Cognitive Psychotherapy” OR “Cognitive Therapy” OR “Cognition Therapy” OR (cognitive* AND behavior* AND therapy*)).

**Inclusion criteria were:** Cross-sectional studies, and articles which had been published in the English language were included. Studies which used only one of the cognitive tests.

**Exclusion criteria:** Clinical trials, cohorts, case-reports, letters to the editors.Two independent researchers collected data regarding first author, country of origin, number of enrolled patients, mean age, applied test for CI evaluation, F/M ratio, mean EDSS, and the number with CI.

**Risk of bias assessment:** We evaluated the risk of potential bias using the Newcastle-Ottawa Scale (NOS) for Assessing the Quality adapted for cross sectional studies (10).

**Statistical analysis:** All statistical analyses were performed using STATA (Version 14.0; Stata Corp LP, College Station, TX, USA). We used random effects model. To determine heterogeneity, Inconsistency (I2) was calculated.

## Results

**Figure 1:**
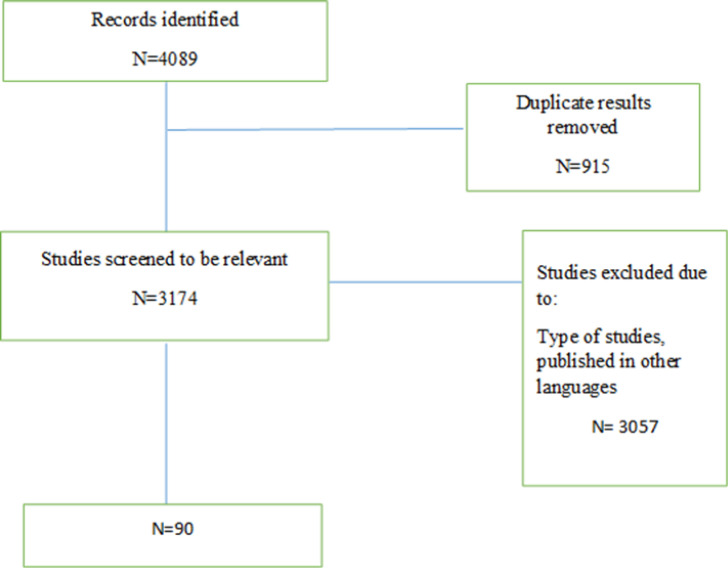
Flow diagram summarizing the selection of eligible studies

**Figure 2:**
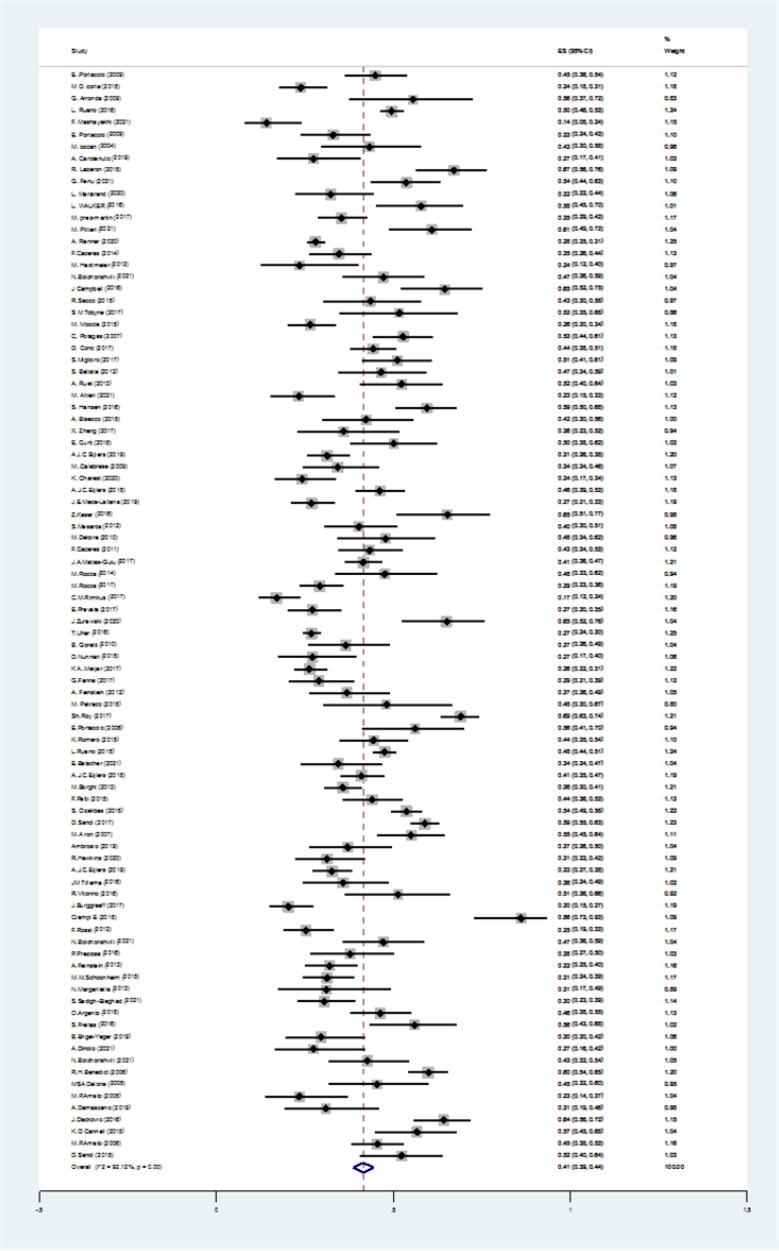
The pooled prevalence of CI using all types of tests

**Figure 3:**
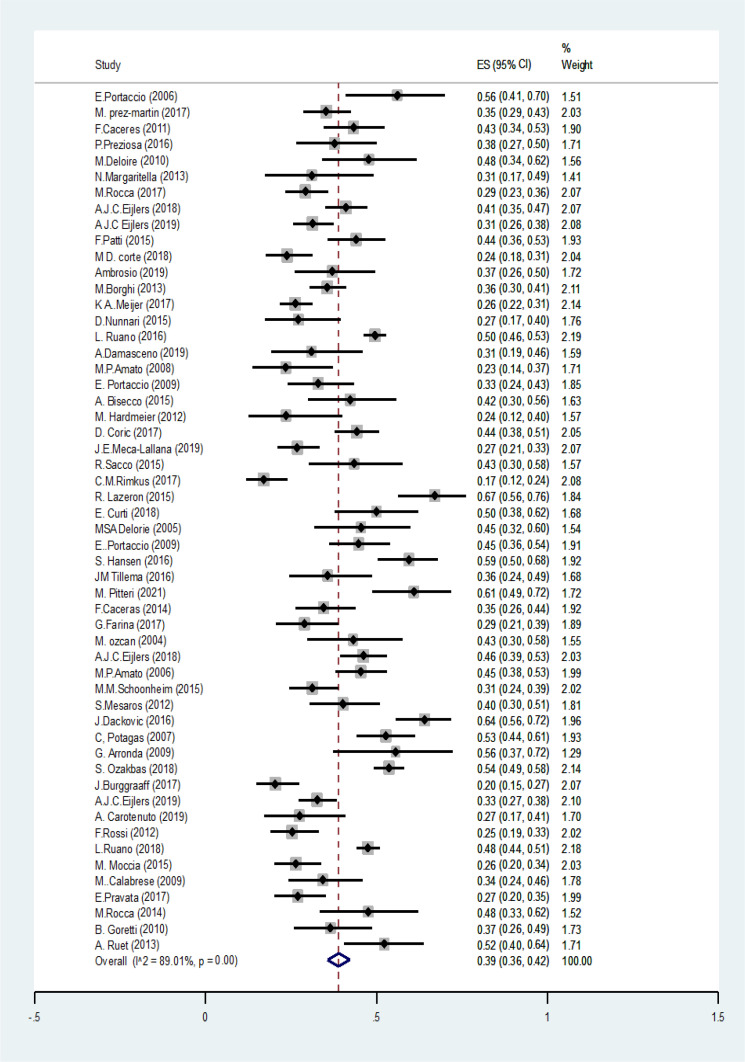
The pooled prevalence of CI using BRB test

**Figure 4:**
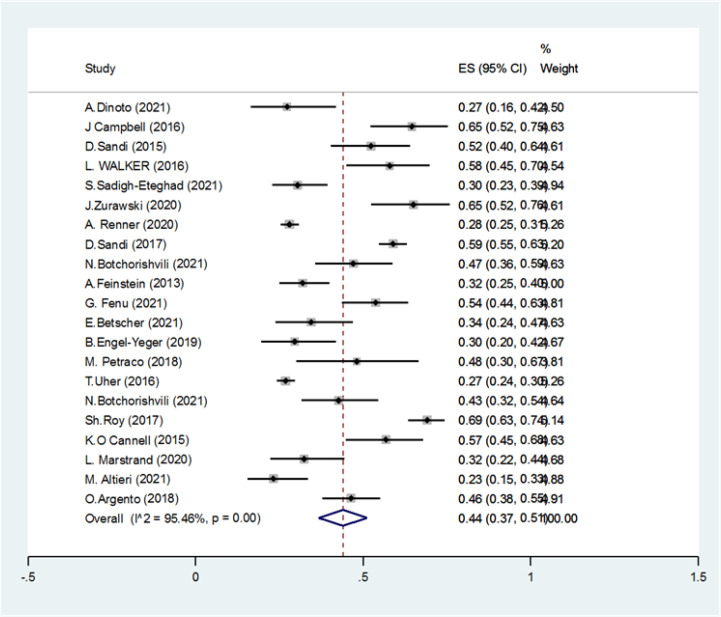
The pooled prevalence of CI using BICAMS

**Figure 5:**
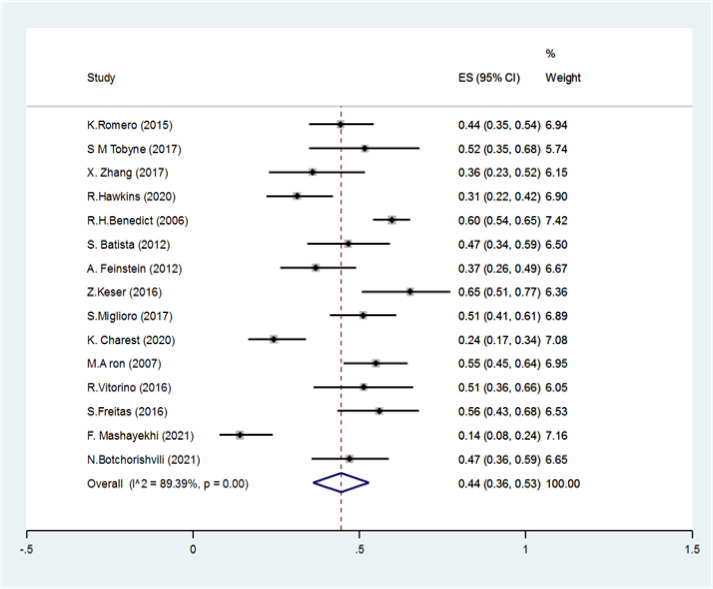
The pooled prevalence of CI using MACFIMS

**Figure 6:**
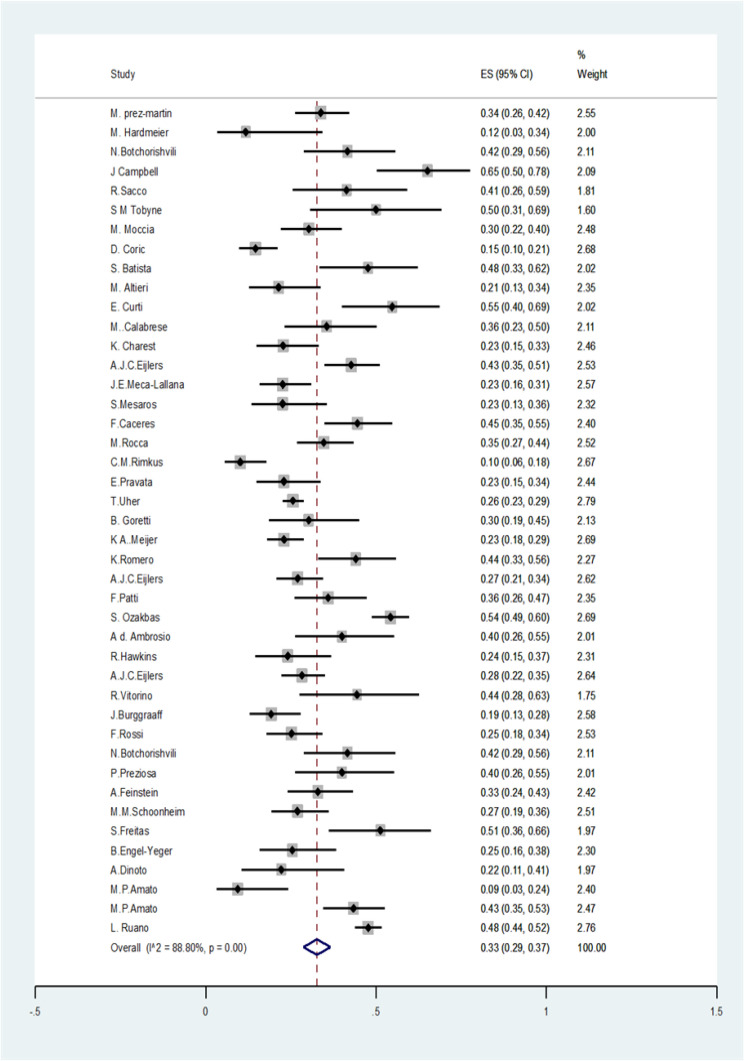
The pooled prevalence of CI in female patients

**Figure 7:**
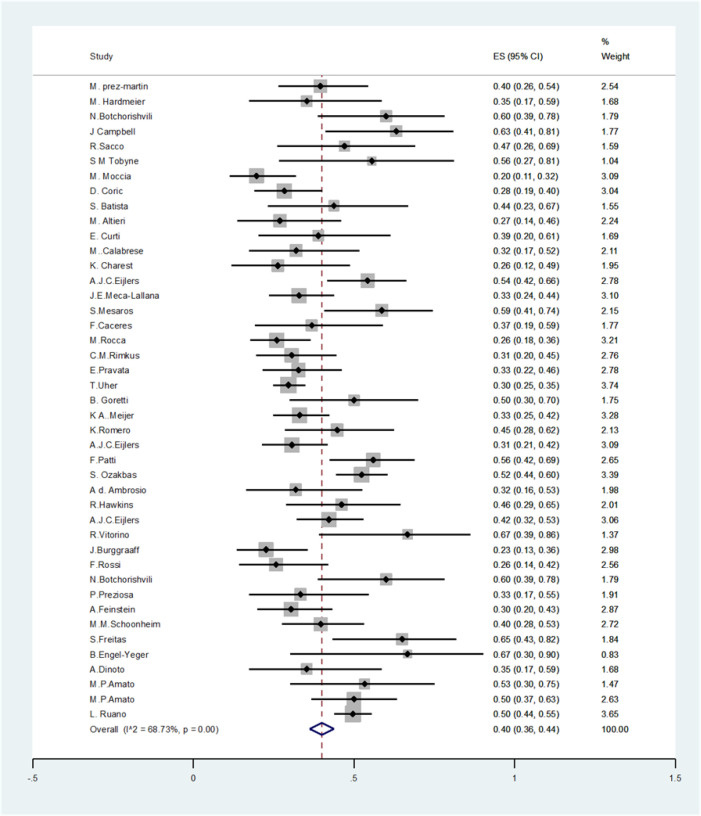
The pooled prevalence of CI in male patients

We found 4089 articles by literature search, after deleting duplicates 3174 remained. Ninety articles remained for meta-analysis (figure 1). The basic characteristics of included studies are summarized in table 1. The pooled prevalence of CI using all types of tests was 41% (95% CI: 38-44%) (I2=91.7%, p<0.001) (Figure 2). The pooled prevalence of CI using BRB test was 39% (95%CI: 36-42%) (I2=89%, p<0.001) (Figure 3). The pooled prevalence of CI using BICAMS was 44% (95%CI: 37-51%, I2=95.4%, Pm<0.001) (Figure 4). The pooled prevalence of CI using MACFIMS was 44% (95% CI: 36-53%) (I2=89.3%, p<0.001) (Figure 5). The pooled prevalence of CI in female patients was 33% (95%CI: 29-37%, I^2^=88%, p<0.001) (Figure 6). The pooled prevalence of CI in male cases was 40% (95%CI: 36-44%) (I^2^=68.7%, p<0.001) (Figure 7).

**Table 1:** Basic characteristics of included studies

**Author/Country/Year**	**Total MS**	**Type of MS**	**F/M MS**	**Age(MEAN)**	**CD diagnosis Criteria**	**Disease duration**	**EDSS**	**Total CD**	**F/M CD**	**CD Age (MEAN)/SD**	**Quality assessment**
E.Portaccio/Italy/2009 (4)	116	RRMS:116	81/35	43.1(9.1)	BRB	15.9 (9.3)	1.7 (1.2)	52	NR	NR	9
M D. corte/Italy/2018 (11)	147	RRMS:128 SPMS:19	107/50	37.76(10.96)	BRB	10.06 (9.41)	3.27 (2.11)	35	NR	38.54 (10.94)	9
G. Arronda/spain/2009 (12)	27	RRMS:13 SPMS:7 PPMS:5 PRMS:2	17/10	44.11(11.45)	BRB	11.41(9.68)	3.7 (0-7.0)	15	NR	NR	8
L. Ruano/Italy/2016 (7)	873	RRMS:759 SPMS:74 PPMS:40	593/280	RRMS:39.9 (10.2) /759 SPMS:51.6 (9.5) /74 PPMS:49.3 (10.9) /40	BRB	RRMS:11.2 (8.4) SPMS:19.4 (10.0) PPMS:12.8 (6.7)	RRMS:2 (1.5-3.5) SPMS:6 (4.5-6.5) PPMS:5.25 (5.0-6.0)	433 RRMS:338 SPMS:59 PPMS:36	F:283/422 M:139/422	43.2 (11.2) /422	9
F. Mashayekhi/Iran/ 2021 (13)	71	RRMS:71	53/18	31.43 (8.75;19-53)	MACFIMS	65.64 (52.44;8-192)-months	1.26 (1.22)	10	NR	NR	8
E. Portaccio/ Italy/2009 (14)	85	RRMS:85	58/27	43 (8.4)	BRB	15.8 (9.6)	1.7 (1.0)	28	NR	NR	9
M. ozcan/Turkey/2014 (15)	44	RRMS:34 SPMS:7 PPMS:3	22/22	NS:34.92 (12.5) /24 HS:40.10 (7.25) /20	BRB	NS: 5.88 (6.35) /24 HS: 6.4 (5.88) /20	NS:1.7 (1.0-7.0) HS:1.5 (1.0-6.5)	19	NR	NR	6
A. Carotenuto /Italy/2019 (16)	51	RRMS:51	26/25	NR	BRB	3 (2.9;0-6)	2.5 (1.0-6.0)	14	NR	NR	10
R. Lazeron/ netherlands/2005 (17)	82	RRMS:31 SPMS:33 PPMS:18	49/33	47 (27-73)	BRB	15.3 (9.3)	1.7 (1.2)	55	NR	NR	9
G. Fenu /Italy/2021 (18)	95	RRMS:95	68/27	43.65 (11.9)	BICAMS	12.1 (7.8)	2 (0-5.5)	51	NR	NR	9
L. Marstrand /Denmark/2020 (19)	65	RRMS:65	41/24	37.2 (8.8;19-56)	BICAMS	3.9 (2.7;1-10)	1.8 (1.2;0-4.0)	21	NR	NR	6
L. WALKER /canada/2016 (20)	57	RRMS:44 SPMS:9 PPMS:4	41/16	45.44 (9.93)	BICAMS	10.11 (7.72)	2.7 (1.85;0-7.0)	33	NR	NR	9
M. prez-martin/spain/2017 (21)	176	RRMS:176	133/43	CI:40.06 (9.31) / 62 CU:44.22 (10.01) / 114	BRB	CI:12.23 (7.17) CU:8.29 (7.17)	CI:2 (1.0-6.5) CI:1.5 (0-6.5)	62	45/17	40.06 (9.31)	9
M. Pitteri/ Italy/2021 (22)	64	RR:61 PPMS:3	54/10	37.3 (11.6)	BRB	3.4 (4.8)	2 (0-4.0)	39 RRMS: 37 PPMS:2	NR	mCI:35.4 (10)/24 sCI:38.1 (3.8)/ 17	8
A. Renner/ Germany/2020 (23)	1094	RRMS:978 SPMS:87 PPMS:29	808/283 out of 1091	T:42.48 (11.20) RRMS:41.47 (10.66) SP:52.28 (11.49) PP:51.66 (11.21)	BICAMS	T:9.61 (7.78) RRMS:9.11 (7.34) SPMS:15.89 (9.43) PPMS:7.74 (8.66)	NR	306 RRMS:253 SPMS:40 PPMS:13	NR	NR	9
F.Caceras /multicenter/2014 (24)	110	RRMS:110	74/36	36.6 (10.6)	BRB	NR	2.07 (0-6.5)	38	NR	NR	9
M. Hardmeier /Netherlands/2012 (25)	34	RRMS:34	17/17	41.4 (0.8)	BRB	8.1 (1.6)	2 (0-4.5)	8	2/6	NR	8
N.Botchorishvili/ Georgia/2021 (26)	68	RRMS: 52 SPMS:12 PPMS:4	48/20	39.2 (9.9)	BICAMS	7 (5.7)	3.3 (1.6)	32 RRMS:21 SPMS:8 PPMS:3	20/12	41.2 (8.9)	8
J Campbell/uk/2016 (27)	62	RRMS:44 SPMS:18	43/19	49.35 (8.88)	BICAMS	12 (8;1-40)	4 (1.0-6.5)	40 RRMS: 27 SPMS: 13	28/12	48.3 (8.33)	8
R.Sacco/ Italy/2015 (28)	46	RRMS:46	29/17	39.6 (7.7)	BRB	11.7 (6.5; 1-25)	2.5 (1.0-6.0)	20	12/8	39.1 (9.8)	7
S M Tobyne/ USA/2017 (29)	31	RRMS:31	22/9	CI:41.33 (3.17) /16 CP:41.19 (2.47) /15	MACFIMS	CI:9.07 (6.49) CP:6.69 (5.99)	CI:2 (1.0-6.5) CP:2 (1.0-4.0)	16	11/5	41.33 (3.17)	8
M. Moccia/ Italy/2015 (30)	155	RRMS:155	99/56	32.1 (8.5)	BRB	3.2 (2.5;0.1-9.1)	1.8 (0.4;1.0-3.0)	41	30/11	35.1 (8.5)	8
C, Potagas /Greece/2007 (31)	127	RRMS:75 SPMS:29 PPMS:23	T:80/47	RRMS:34.3 (8.9) SPMS:42.0 (8.5) PPMS:42.8 (9.9)	BRB	RRMS:6.2 (4.9) SPMS:15.3 (7.9) PPMS:4.7 (5.3)	RRMS:1.9 (1.6) SPMS:5.6 (1.3) PPMS:4.7 (5.3)	T:67 RRMS:30 SPMS:24 PPMS:13	NR	NR	8
D. Coric/netherlands /2017 (32)	217	RRMS:133 SPMS:56 PPMS:28	150/67	54.30 (9.96)	BRB	20.34 (6.99)	4 (1.0-8.0)	96	22/19	57.66 (9,59)	9
S.Miglioro/Italy/2017 (33)	92	RRMS:92	64/28	41.5 (10.7)	MACFIMS	9.5 (0.3-30.1)	1 (0-2.5)	47	NR	NR	9
S. Batista/USA /2012 (34)	58	RRMS:50 SPMS:5 PPMS:1	42/16	43.5 (6.5)	MACFIMS	9.6 (6.6)	2.5 (0-4.5)	27	20/7	43.4 (6)	8
A. Ruet /France/2013 (35)	65	RRMS:54 PMS:11	45/20	39 (10.4)	BRB	31.2 (38.2)	2 (0-6.0)	34	NR	NR	8
M. Altieri/ (36) Italy/2021	82	RRMS:82	56/26	45.8 (11)	BICAMS	3.9 (2)	2.8 (1.8)	19	12/7	NR	9
S. Hansen /Germany/2016 (37)	116	RRMS:90 SPMS:26	77/39	42.8 (11.5)	BRB	T:10.5 (8.5) RRMS:9 (7.3) SPMS:16.5 (10.3)	T:2.5 (1.7) RRMS:2 (1.3) SPMS:4 (1.4)	69	NR	NR	8
A. Bisecco /MULTICENTER/2015 (38)	52	RRMS:52	33/19	40.3 (8.5)	BRB	8.4 (2-33)	2 (0-6)	22	15/7	43.4 (8.6)	7
X. Zhang /China/2017 (39)	39	RRMS:39	23/16	38.26 (9.05)	MACFIMS	92.33 (71.54)-months	2.24 (1.58)	14	NR	38.86 (9.02)	8
E. Curti/ Italy/2018 (40)	60	RRMS:47 SPMS:11 PPMS:2	42/18	39.5 (11.13)	BRB	101.2 (86.87)-months	2 (1.0-6.5)		23/7	29.5 (10.7)	7
A J.C Eijlers/ Netherlands/2019 (41)	230	RRMS:179 SPMS:32 PPMS:19	156/74	47.66 (11.07)	BRB	14.83 (8.48)	3 (0-8.0)		NR	NR	10
M..Calabrese/ Italy/2009 (42)	70	RRMS:70	45/25	34.8 (15-55)	BRB	8.4 (1-18)	CI:2.9 (1.0-5.0) CU:1.9 (1.0-4.5)		16/8	36.1	7
K. Charest /canada/2020 (43)	91	RRMS:76 SPMS:15	79/19	CI:50.3 (10.4) CU:49.5 (11.8)	MACFIMS	CI:9.9 (6.7) CU:10.5 (7.7)	CI:2-1 (2.2) CU:1.7 (1.9)		18/5	50.3 (10.4)	7
A.J.C.Eijlers /netherlands/2018 (44)	197	RRMS:141 SPMS:30 PPMS:26	138/59	No GM CU:48.51 (9.74) /90 No GM CI:51.45 (12.39) /42 GM CU:41.32 (9.35) /16 GM CI:48.13 (10.09) /49	BRB	No GM CU:13.47 (7.66) /90 No GM CI:13.73 (7.78) /42 GM CU:13.61 (8.05) /16 GM CI:16.02 (9.86) /49	No GM CU:3 (0-8.0) /90 No GM CI:3.5 (2.0-8.0) /42 GM CU:2.75 (1.5-7.5) /16 GM CI:4 (0-8) /49		59/32	No GM CI:51.45 (12.39) /42 GM CI:48.13 (10.09) /49	7
J.E.Meca-Lallana /Spain/2019 (45)	194	RRMS:174 PMS:10	115/79	42.3 (9)	BRB	9.9 (7.1)	2 (1.0-3.5)	52 RRMS:44 PMS:8	26/26	44.1 (8.9)	9
Z.Keser/USA/2016 (46)	46	RRMS:38 SPMS:8	NR	40.8 (11.26;18-56)	MACFIMS	13.29 (9.21)	3.51 (2.03;0-7)	30	NR	42.32 (11.33)	7
S.Mesaros/ Serbia/2012 (47)	82	RRMS:20 SPMS:19 PPMS:23 BMS:20	53/29	44 (22-60)	BRB	12.1 (1-40)	3.4 (0-8.5)	33	12/17 out of 29	49	9
M.Deloire/ france/2010 (48)	46	RRMS:46	36/10	38.6 (8.7)	BRB	23.5 (27.1)-months	2 (0-5.5)	22	NR	NR	8
F.Caceres /argentina/2011 (49)	111	RRMS:93 SPMS:10 PPMS:4 RPMS:4	92/19	CI:41.5 (11.2) /48 CU:40.3 (11.4) /63	BRB	7.4 (7)	CI:3.79 (1.99) CU:2.73 (1.56)	48	41/7	41.5 (11.2)	8
M.Rocca/ MULTICENTER/2014 (50)	42	RRMS:42	23/19	39.6 (8.5;24-55)	BRB	7.7 (2-15)	2 (1.0-4.0)	20	NR	42.6 (8.1)	8
M.Rocca/ Italy/2017 (51)	202	RRMS:119 SPMS:41 PPMS:13 BMS:29	121/81	RRMS: 37.5 (18.9-60.9) /119 SPMS:48.4 (26.0-66.1) PPMS:52.2 (42.2-67.9) BMS:44.7 (27.1-66.4)	BRB	12.1 (0.1-44.7)	2 (0-8.5)	59 RRMS:29 SPMS:14 PPMS:6 BMS:10	42/21	45.9 (20.9-67.9)	9
C.M.Rimkus /Netherlands/2017 (52)	147	RRMS:124 SPMS:8 PPMS:15	98/49	41.6 (8.5)	BRB	7.5 (2.3)	2.8 (1.5)	25 RRMS:14 SPMS:3 PPMS:8	10/15	47 (8.2)	9
E.Pravata/Italy/ 2017 (53)	126	RRMS:87 SPMS:14 PPMS:4 BMS:21	74/52	37.4 (11.7)	BRB	11.5 (0.8-36)	1.5 (0-8.0)	34	17/17	40.7 (11.1)	9
J.Zurawski /USA/2020 (54)	60	RRMS:49 SPMS:10 PPMS:1	41/19	49.2 (10.6)	BICAMS	9.2 (9)	2.2 (1.2)	39	NR	NR	7
T.Uher/chzech/2016 (55)	1052	RRMS:1052	734/318	CI:39.6 (8.8) /282 CU:37.6 (8.8) /770	BICAMS	CI: 11.6 (7.6) CU: 8.8 (6.7)	CI: 3.4 (1.4;0–6.5) CU: 2.2 (1.1;0–6.5)	282	188/94	39.6 (8.8)	9
B. Goretti/ Italy/2010 (56)	63	RRMS:55SPMS:8	43/20	42.6 (10.1)	BRB	14.7 (10.8)	2.2 (1.7)	23	13/10	45.9 (10.8)	9
D.Nunnari/ Italy/2015 (57)	59	RRMS:52/60 PPMS:8/60	38/21 out of 60	39.3 (10.6) /60	BRB	6.3 (5.2)/60	2.4 (1.4) /60	16	NR	NR	9
K A..Meijer /netherlands/2017 (58)	332	RRMS:243 SPMS:53 PPMS:36	226/106	48.1 (11)	BRB	CI: 18 (5-46) MCI: 13 (5-35) CU: 10 (5-34)	CI: 4 (2-8) MCI: 3 (0-8) CU: 3 (0–8)	142 RRMS:95 SPMS:32 PPMS:25	52/35	51 (10.7)	8
G.Farina/Italy/ 2017 (59)	90	RRMS:71 SPMS:19	62/28	42.5 (10.7; 17-69)	BRB	7.9 (5.7;0-22)	2.0 (1.0-8.0)	26	NR	NR	9
A. Feinstein/ Canada/2012 (60)	65	RRMS:38 SPMS:18 PPMS:9	44/21	45.75 (8.86)	MACFIMS	9.54 (7.15)	3.59 (2.59)	24	NR	NR	9
M. Petraco/ USA/2018 (61)	25	PPMS:25	14/11	51.2 (10.41)	BICAMS	9.04 (4.64)	NR	12	NR	NR	7
Sh.Roy/USA/2017 (62)	275	RRMS:199 SPMS:66 PPMS:10	202/73	47.41 (10.76)	BICAMS	10.31 (8.74)	3 (0-8.0)	190	NR	NR	9
E.Portaccio/Italy/ 2006 (63)	41	RRMS:41	30/11	35.1/34 (8.6;20-55)	BRB	4 (2.8;0.5-10)	1.5 (0.6;1.0-4.0)	23	NR	NR	8
K.Romero/Canada/2015 (64)	97	RRMS:81 SPMS:8 PPMS:6 BMS:2	68/29	CI:40.86 (10.76) /43 CU:42.87 (12.57) /54	MACFIMS	CI:9.49 (7.22) CU:9.14 (7.53)	CI:2.71 (2.13) CU:2 (1.97)	43 RRMS:36 SPMS:5 PPMS:1 BMS:1	30/13	40.86 (10.76)	9
L.Ruano/Italy/2018 (65)	831	RRMS:NR SPMS:NR	569/262	Adult onset:41.9 (35.0-49.2) /712 Pediatric-onset:29.7 (24.4-37.9) /119	BRB	Adult onset:9.2 (4.7-16.6) Pediatric-onset:13.2 (8.1-21.5)	Adult onset:2 (1.5-4.0) Pediatric-onset:2.5 (1.5-4.0)	395	NR	NR	9
E.Betscher/ Poland/2021 (66)	61	RRMS:45 SPMS:12 PPMS:4	45/16	39 (28-49)	BICAMS	7 (3-13)	3.5 (2.0-4.5)	21	NR	NR	9
A.J.C.Eijlers/ Netherlands/2018 (67)	234	RRMS:181 SPMS:33 PPMS:20	159/75	47.61 (11.02)	BRB	14.8 (8.4)	3 (0-8.0)	96	43/23	49.77 (10.80)	9
M.Borghi/Italy/2013 (68)	303	RRMS:303 SPMS:21 PPMS:9 RPMS:6	212/91	43.07 (10.79)	BRB	10.87 (7.26)	2.43 (1.92)	108	NR	NR	9
F.Patti/Italy/2015 (69)	125	RRMS:103 SPMS:14 PPMS:8	75/50	41.4 (10.3)	BRB	8 (3.3)	2.2 (1.9;0-8.0)	55	27/28	46.3 (10.2)	8
S. Ozakbas/ Turkey/2018 (70)	462	RRMS:462	317/145	T:35.3 (9.39)	BRB	7.75 (5.9)	1.9 (1.31)	248	172/76	37.32 (9.31)	8
D.Sandi/ Hungary/2017 (71)	525	RRMS:525	NR	44.91 (11.65)	BICAMS	13.71 (8.14)	2 (6.5;2.0)-range;IQR	309	NR	NR	9
M.A ron/USA/2007 (72)	100	RRMS:70 SP:30	78/22	44.61 (8.39)	MACFIMS	NR	NR	55	NR	NR	10
A d. Ambrosio/Multicenter/2019 (73)	62	RRMS:62	40/22	39.5 (8.5)	BRB	8.2 (6.3)	2 (0-6.0)	23	16/7	43.3 (8.3)	8
R.Hawkins/ Canada/2020 (74)	80	RRMS:80	54/26	51.8 (8.6)	MACFIMS	15.8 (8.9)	4.3 (2.3)	25	13/12	49.7 (7.9)	9
A.J.C.Eijlers/ Netherlands/2019 (41)	267	RRMS:197 SPMS:47 PPMS:23	184/83	47.9 (10.8)	BRB	14.7(8.5)	CI:4 (2-8) CU:3 (0-8.0)	87 RRMS:49 SPMS:26 PPMS:12	52/35	51.1 (10.7)	8
JM Tillema /MULTICENTER/2016 (75)	56	RRMS:56	35/21	39.2 (8.85)	BRB	CI:10.8 (8.4) CU:6.8 (4.4)	CI:2.5 (1.3) CU:1.9 (1.0)	20	NR	43.3 (8.9)	8
R.Vitorino/ USA/2016 (76)	39	RRMS:39	27/12	CI:48.1 (4.7) /20 CU:46.4 (7.2) /19	MACFIMS	CI:11.6 (4.9) CU:11.8 (5.4)	CI:2.6 (0.7) CU:1.8 (0.7)	20	12/8	48.1 (4.7)	9
J.Burggraaff/ Netherlands/2017 (77)	157	RRMS:133 SPMS:9 PPMS:15	104/53	41.1 (8.2)	BRB	7.5 (2.2)	2.5 (0-8.0)	32 RRMS:21 PMS:11	20/12	42.9 (8.5)	9
F.Rossi/Italy/2012 (78)	142	RRMS:142	107/35	39.4 (9.1;18.5-57.5)	BRB	11 (9.8)	1.8 (1.2)	36	27/9	42.8 (9.12)	8
N.Botchorishvili/ Gorgia/2021 (26)	68	RRMS:52 SPMS:12 PPMS:4	48/20	39.2 (9.9)	BICAMS	7 (5.7)	3.3 (1.6)	32	20/12	41.2 (8.9)	8
P.Preziosa/ Multicenter/2016 (79)	61	RRMS:61	40/21	39.7 (8.5)	BRB	8.2 (6.4)	1.5 (0-6.0)	23	16/7	43.3 (8.4)	8
A.Feinstein/ Canada/2013 (80)	144	RRMS:79 SPMS:45 PPMS:20	88/56	46.8 (10.32)	MACFIMS	11.45 (8.62)	CI:5.1 (2.3) CU:3.2 (2.5) CP:3.3 (2.4)	46 RRMS:20 SPMS:18 PPMS:8	29/17	47 (10.0)	9
M.M.Schoonheim/ Netherlands/2015 (81)	157	RRMS:133 SPMS:9 PPMS:15	104/53	CU:40.13 (8.06) /108 MCI:41.96 (8.91) /22 SCI:45.70 (9.27) /27	BRB	MCI:7.57 (2.43) SCI:7.42 (2.09) CU:7.49 (2.21)	MCI: 2.5 (1.0-8.0) /22 SCI:4 (2-7.5) /27 CU:2 (0-8.0) /108	49 RRMS:35 SPMS:6 PPMS:8	28/21	MCI: 41.96 (8.91) /22 SCI:45.7 (9.27) /27	9
N.Margaritella/ Italy/2013 (82)	29	BMS:29	25/4	31.7 (8.8)	BRB	4.1 (4.4)	0.7 (0.6)	9	NR	NR	7
S.Sadigh-Eteghad/ Iran/2021 (83)	115	RRMS:87 SPMS:21 PPMS:7	80/35	34.13 (9.8)	MACFIMS	86.70 (64.52)-months	2 (1.94)	35 RRMS:18 SPMS:12 PPMS:5	NR	28.68 (8.68)	9
O.Argento/ Italy/2018 (84)	123	RRMS:NR SPMS:NR	77/46	43.77 (10.18)	MACFIMS	RRMS:9.21 (7.51) SPMS:18.10 (9.48)	RRMS:2.4 (1.03) SPMS:4.48 (1.10)	57	NR	NR	7
S.Freitas/ Portugal/ 2016 (85)	59	RRMS:51 SPMS:8	39/20	37.2 (7.58)	BICAMS	10.39 (6.55)	2.5 (1.4)	33	20/13	39.91 (7.03)	8
B.Engel-Yeger/ USA/2019 (86)	61	RRMS:57 SPMA:4	55/6	CU:48.26 (10.07) /43 CI:50.11 (8.21) /18	BICAMS	CI:241.6 (111.3)-months CU:170.8 (96.3)=-months	NR	18 RRMS:16 SPMS:2	14/4	50.11 (8.21)	8
A.Dinoto/Italy/2021 (87)	44	RRMS:44	27/17	39.36 (11.10)	BICAMS	3 (0-10)	1 (1-4.0)	12	6/6	40.16 (12.33)	8
N.Botchorishvili/ Gorgia/2021 (26)	68	RRMS:52 SPMS:12 PPMS:4	48/20	39.2 (9.9)	BICAMS	7 (5.7)	3.3 (1.6)	29 RRMS:18 SPMS:8 PPMS:3	NR	NR	8
R.H.Benedict/ USA/2006 (88)	291	RRMS:200 SPMS:78 PPMS:7 PRMS:6	227/64	45.4 (8.9)	MACFIMS	NR	3 (1.8) /186	174	NR	NR	9
MSA Delorie/ France/2015 (89)	58	RRMS:44	44/14	37.34 (9.17)	BRB	24.33 (26.49)-months	2 (0-6.5)	44	NR	NR	8
M.P.Amato/ Italy/2008 (90)	47	BMS:47	32/15	46.4 (8.4)	BRB	22.5 (6.0)	1.3 (0.9)	11	3/8	49.9 (7.2)	8
A.Damasceno/ Brazil/2019 (91)	42	RRMS:42	32/10	30.52 (6.6)	BRB	6.4 (4.94)	2.25 (0-4.0)	13	NR	NR	8
J.Dackovic/Serbia /2016 (92)	131	RRMS:65 SPMS:31 PPMS:35	86/45	RRMS:37.8 (11.0) /65 SPMS:46.8 (9.1) /31PPMS:46.8 (10.1) /35	BRB	RRMS:8.30 (10.1) SPMS:18.6 (8.7) PPMS:6.3 (5.1)	RRMS:3 (1.0-4.0) SPMS:6.5 (4.0-8.5) PPMS:5.5 (5.5-7.5)	84 RRMS:24 SPMS:30 PPMS:30	NR	NR	9
K.O Cannell/ Ireland/2015 (93)	67	RRMS:47 SPMS:19 PPMS:1	49/18	43.9 (12.1)	BICAMS	RRMS:10.2 (8.4) SPMS:20.6 (10.2) PPMS:17	RRMS:1.8 (0.9) SPMS:5.7 (1.4) PPMS:7.0	38 RRMS:23 PMS:15	NR	NR	7
M.P.Amato/ Italy/2006 (94)	163	BMS:163	113/50	44.5 (7.7)	BRB	20.8 (5.3)	1.8 (0.8)	74	49/25	45.5 (8.1)	9
D.Sandi/ Hungary/2015 (95)	65	RRMS:65	49/16	41.9 (8.9)	BICAMS	11.1 (7.6)	2.5 (1.8)	34	NR	NR	8

CD: Cognitive dysfunction, RRMS: Relapsing-remitting, SPMS: Secondary progressive MS, PPMS: Primary progressive MS, BMS: Benign MS, PRMS: Progressive relapsing MS, BRB: Brief repeatable battery, MACFIMS: Minimal assessment of cognitive function in MS, BICAMS: Brief international cognitive assessment for MS, GM: Gray matter, NS: Nonsmokers, HS: Heavy smokers, CI: Cognitive impairment, CU: Cognitive unimpairment, mCI: Mild Cognitive impairment, sCU: Sever cognitive impairment, T: Total.

## Discussion

The result of this sytematic review and meta-analysis show that the pooled prevalence of CI in patients with MS is 41%, while the prevalence in included studies ranged between 14-69%.When we analyzed CI based on the applied test, the proportion of CI was higher by using MACFIMS and BICAMS (44% vs 39% by BRB).

We also found that the pooled prevalence of CI in male patients was 40% while this rate was 33% in female ones which was not correlated with demographic, psychiatric or neurologic variables. They suggested that the size or location of the lesions plays a role in CI development in men (96). They also found that memory and visuospatial construction aspects were mostly affected in men than women (96).

Beatty and Aupperle found that male patients with MS suffer more from CI than female ones and Campbell et al. enrolled 62 patients with MS using BICAMS test for cognition evaluation. They found that 65% of enrolled cases had CI which was associated with lower quality of life and unemployment. Overall, CI is common in MS and patients with progressive form of the disease suffering more from this complication.

 In a cohort study, it was shown that disability status, course of the disease, and advanced age are more predictors of CI in patients with MS (97). Patients with secondary progressive form are at two fold higher risk of developing CI than RR form (12). In another study, Coric et al. enrolled 217 patients with MS and found that 44% had CI using BRB test (13). Zhang et al. applied MACFIMS test and reported CI in 14 out of 39 enrolled cases (39).

CI is common in MS and also neuromyelitis optical spectrum disorders (NMOSD). In a recent systematic review and meta-analysis, the pooled prevalence of CI in NMOSD estimated as 44% which is similar to our findings (44% vs 41%) (98). CI could have been detected in early stages of the disease or in patients with clinically isolated syndrome (CIS) which indicates that CI starts before definite diagnosis of MS (99). On the other hand, psychological problems such as depression, fatigue, and anxiety are common in MS, psychological factors could affect cognition in MS (1, 100). Other factors such as disease duration, progression of the disease, and gray matter atrophy are considered as important items in developing CI (101). In a multivariate analysis, Ruano et al. investigated that advanced age and physical disability are significant predictors of CI in MS (7).

Physicians should consider CI and its evaluation in patients with MS as it has a wide range of consequences for patients. Improving psychological well-being (treating depression and anxiety), sleep quality improvement, attending cognitive rehabilitation courses, administering disease modifying therapies (DMTs) such as interferon beta (IFNb), natalizumab will impact positively on cognition status of patients with MS (102). Other strategies such as cognitive–behavioral therapy, transcranial direct current stimulation, strategy-oriented neuropsychological rehabilitation, and physical exercise training are considered as positive therapies for cognitive improvement (103).

The wide range of prevalence of CI in included studies could be due to different inclusion criteria of the patients, administration of various tests, ethnicity, and including patients with no similar clinical course. On the other hand, authors used various scoring systems (2 or 3 SD) for defining CI. This systematic review has few strengths. First, we included 90 studies. Second, we analyzed based on different applied tests. 

Third, we estimated CI based on sex. It seems that the pooled prevalence of CI based on various tests ranges between 39%-44% which highlights the importance of cognitive evaluation by physicians. The pooled prevalence of cognitive impairment in patients with MS is estimated as 41%, so CI should be considered by clinicians.
